# How do perceived and objective measures of neighbourhood disadvantage vary over time? Results from a prospective-longitudinal study in the UK with implications for longitudinal research on neighbourhood effects on health

**DOI:** 10.1371/journal.pone.0231779

**Published:** 2020-04-16

**Authors:** Alexa R. Yakubovich, Jon Heron, David K. Humphreys

**Affiliations:** 1 Department of Social Policy and Intervention, University of Oxford, Oxford, England, United Kingdom; 2 MAP Centre for Urban Health Solutions, Li Ka Shing Knowledge Institute, St. Michael's Hospital, Unity Health Toronto, Toronto, Ontario, Canada; 3 Department of Population Health Sciences, Bristol Medical School, University of Bristol, Bristol, England, United Kingdom; 4 MRC Integrative Epidemiology Unit at the University of Bristol, Bristol, England, United Kingdom; King Fahd University of Petroleum & Minerals, SAUDI ARABIA

## Abstract

**Background:**

Theories of health outcomes often hypothesize that living in more socially and economically disadvantaged neighbourhoods will lead to worse health. Multiple measures of neighbourhood disadvantage are available to researchers, which may serve as better or worse proxies for each other across time. To inform longitudinal study design and interpretation we investigated how perceived and objective measures of neighbourhood disadvantage vary over time and the factors underlying this variation.

**Methods:**

Data were from 8,918 mothers with at least three time-points of neighbourhood data in the Avon Longitudinal Study of Parents and Children in the UK. We analyzed measures of objective (Indices of Multiple Deprivation) and perceived (neighbourhood quality, social cohesion, and stress) exposure to neighbourhood disadvantage at 10 time-points over 18 years. We used group-based trajectory modelling to determine the overlap in participants' trajectories on the different measures and evaluated the baseline factors associated with different perceived trajectories over time.

**Results:**

There was evidence of heterogeneity in both perceived and objective measures of neighbourhood disadvantage over time (e.g., on the objective measure, 5% of participants moved to more deprived neighbourhoods, 11% moved to less deprived neighbourhoods, 20% consistently lived in deprived neighbourhoods, and 64% consistently lived in non-deprived neighbourhoods). Perceived social cohesion showed the weakest relationship with exposure to objective neighbourhood deprivation: most participants in each trajectory group of objective neighbourhood deprivation followed non-corresponding trajectories of perceived social cohesion (61–80%). Accounting for objective deprivation exposure, poorer socioeconomic and psychosocial indicators at baseline were associated with following more negative perceived neighbourhood trajectories (e.g., high neighbourhood stress) over time.

**Conclusion:**

Trajectories of perceived and objective measures of neighbourhood disadvantage varied over time, with the extent of variation depending on the time point of measurement and individual-level social factors. Researchers should be mindful of this variation when choosing and determining the timing of measures of neighbourhood disadvantage in longitudinal studies and when inferring effect mechanisms.

## Introduction

Etiological theories of health typically hypothesize structural and community contexts as upstream determinants–including, for instance, that living in more socially disadvantaged or disorganized neighbourhoods will lead to worse health outcomes.[1–3] To investigate these hypothesized neighbourhood effects there is growing consensus that longitudinal studies are needed to account for the duration and timing of neighbourhood exposures.[4–8] A fundamental issue in these studies is how to measure neighbourhood disadvantage.[9] This is even more complex in longitudinal studies, where the extent of variation between different measures of the neighbourhood may itself change over time, with implications for study design and interpretation.[10]

So-called *objective* measures of neighbourhoods capture distinct aspects of the physical and social environment (e.g., available facilities, crime rates) within defined physical areas (e.g., census units).[9] In theory, this allows for conclusions regarding whether and how these features or phenomena relate to health outcomes, informing intervention and policy targets. In practice, these measures are often routinely collected and convenient to use. However, objective measures fail to capture experiential or relational aspects of the neighbourhood environment, which are often hypothesized as part of the mechanisms of neighbourhood effects.[9,11,12] For instance, one of the most prolific neighbourhood theories posits that collective efficacy–or social cohesion between members of the neighbourhood and their perceived willingness to intervene on behalf of the 'common good'–mediates the positive relationship between neighbourhood disadvantage and health and criminological outcomes.[1,13] Measuring these social constructs requires reports from neighbourhood members typically not collected in routine data. The definitions of neighbourhoods used in objective measures also do not always correspond with individuals' own conceptions of their neighbourhoods.[11,14–16] These varying definitions of neighbourhoods can dramatically alter study results, known as the modifiable areal unit problem.[17]

Perceived measures of neighbourhoods can address these limitations by allowing participants to report on the social contexts of their neighbourhoods that are more difficult (or impossible) to capture by objective measurement. Participants can also report on the physical components of their neighbourhoods as they conceive and experience them. Yet matching participants' perceptions or definitions of their neighbourhoods to fixed or consistent areas for intervention design is resource intensive and sometimes impossible in secondary data analysis.[14,15,18] Likewise, perceived neighbourhood measures may capture individual-level characteristics, even when aggregated, that confound neighbourhood effects.[11,19]

The strengths and weakness of these different neighbourhood measures mean researchers must make a choice in study design informed by the varying constructs being captured, the research question or hypothesis, and available resources. Perceived measures of neighbourhood disadvantage are often thought to correlate with objective measures. This assumption is made both in theories like collective efficacy theory which directly hypothesize mediating mechanisms as well as the general expectation that perceptions of the environment will be influenced by and serve as reasonable proxies for objective conditions.[2,13,20,21] To our knowledge, despite the recognized need for longitudinal studies of neighbourhood effects, no longitudinal study has investigated the extent and determinants of variation between individuals' perceptions of their neighbourhood and their objective exposure to neighbourhood disadvantage over time (and the life course). This is important for informing the selection and timing of measures in longitudinal studies on the effects of neighbourhood disadvantage and how studies that use different measures should be interpreted.

Available studies have largely focused on the relatively low point-in-time agreement between perceived and objective measures of neighbourhood characteristics specific to physical activity (e.g., walkability).[22–29] Variation in people's perceptions of the built environment have further shown to be correlated with sociodemographic and psychosocial factors (e.g., socioeconomic status, depression). Within and beyond the physical activity literature, studies have also demonstrated low to moderate correspondence between police-reported and perceived crime, with unique impacts on health and wellbeing.[26,30–35] A more limited number of studies have found differential impacts of point-in-time perceived and objective measures of broader neighbourhood conditions (e.g., neighbourhood deprivation) on health and behavioural outcomes.[36–41] To address the existing evidence gaps in the longitudinal neighbourhood literature we had two aims: (1) summarize the dynamic nature of the relationship between perceived and objective measures of exposure to neighbourhood disadvantage and (2) determine how social factors are related to variations in neighbourhood perceptions over time.

## Method

Data were from the Avon Longitudinal Study of Parents and Children (ALSPAC), an ongoing prospective-longitudinal study. All pregnant women resident in one of three health districts in the former county Avon in the United Kingdom (UK) due between 1 April 1991 and 31 December 1992 were eligible to participate.[42,43] Initially, 14,541 pregnant women (and their eventual babies) were enrolled. When the children of enrolled mothers were age 7 eligible mothers not enrolled were contacted, increasing the sample to 15,454 mothers (76% of all eligible) with 14,901 babies alive at age 1. The current study uses data from the sample of participating mothers whose children were also enrolled in the study across ten time points (Table 1). The time points in ALSPAC are labelled by the age of the enrolled children, which we maintain in the current study. The ALSPAC Ethics and Law Committee and Local Research Ethics Committees provided ethical approval. Informed consent was obtained from participants following the recommendations of the ALSPAC Ethics and Law Committee at the time.

**Table 1:** Timing of measurement for neighbourhood variables.

	Time point									
**Age of mother's eligible child**	**0**	**1.75**	**3**	**5**	**7**	**10**	**12**	**14**	**16.5**	**18**
**Year**	**1990**	**1992**	**1993**	**1994**	**1997**	**1999**	**2002**	**2006**	**2007**	**2010**
**N with any neighbourhood data**	**13,909**	**10,412**	**9,739**	**9,510**	**9,106**	**9,060**	**7,219**	**5,787**	**4,894**	**5,287**
**Objective neighbourhood deprivation**										
**Perceived neighbourhood variables**:										
**Neighbourhood opinion**										
**Social cohesion**										
**Neighbourhood stress**										

Shaded box indicates variable was measured at time point indicated.

### Measures

#### Objective neighbourhood measure

We measured participants' exposure to objective neighbourhood environments using the 2010 Indices of Multiple Deprivation.[44] These were available for ten time points from baseline (pregnancy) to age 18 (Table 1) and were based on the enrolled children's addresses, updated annually by ALSPAC for regular communications and questionnaires. This requires the assumption that the addresses of mothers and their enrolled child were the same, which is reasonable in most cases given the children's ages during the study period (0–18 years) and the fact that mothers were typically their primary caregivers.

The Indices of Multiple Deprivation are composed of 38 indicators across seven domains of deprivation (income, employment, education, health, crime, housing, and living environment) (Table A1, S1 Appendix). Each lower-layer super output area (LSOA) in England–small census areas of ~1500 residents or 650 households that approximate residential neighbourhoods–is assigned a domain-specific and total deprivation rank score relative to all other neighbourhoods.[45] We had access to participants' quintile ranks for each domain and the total deprivation index–determined by ALSPAC to protect anonymity–so that at each time point (Table 1), we knew the quintile of deprivation each participant's residential neighbourhood fell into relative to all other neighbourhoods in England (i.e., not just within ALSPAC). Due to the underlying exponential distribution of the deprivation scores, there is less variation in deprivation between the less deprived quintiles (e.g., quintiles 1 to 3) compared to the most deprived: for further details, see Fig A1 (S1 Appendix) and the measure's technical report.[46] To account for this, we computed a binary variable at each time-point, where 1 = deprivation quintiles 4 and 5 (i.e., living in the 40% most deprived neighbourhoods in England) and 0 = otherwise. This allowed for a comparison of exposure to more versus less objective neighbourhood deprivation while maintaining response variation (the proportion of participants in quintile 5 decreased to ~6% over time). We use a stricter dichotomization (quintile 5 versus all others) in sensitivity analyses.

#### Perceived neighbourhood measures

Table A2 (S1 Appendix) shows the items in each composite variable, adapted for use in ALSPAC[43] and similar to those used in other UK cohort studies.[47] Mothers rated their neighbourhood's quality, where 1 = very good place to live, 2 = fairly good place to live, 3 = not a very good place to live, or 4 = not at all a good place to live. At later time points, few mothers scored their neighbourhoods as 'not a very good place to live' (e.g., at the age 18 time-point, n = 42) or 'not at all a good place to live' (n = 14). To maximize cell counts, *suboptimal neighbourhood quality* was dichotomized as 0 = very good place (1), 1 = otherwise (2–4). *Poor social cohesion* was measured as the mean score on seven items (e.g., how often neighbourhood people visit the home) on a 5-point scale (0 = always, 4 = never) (α = .78-.82 at each time). *Neighbourhood stress* was the mean score on 11 items (e.g., size of the problem of noise from other homes) on a 3-point scale (0 = no problem or opinion, 2 = serious problem) (α = .78-.82). At the age 18 time-point, the neighbourhood stress items changed slightly (e.g., badly fitted doors/windows removed; traffic added) (Table A2, S1 Appendix). All variables were measured at seven times apart from neighbourhood stress, which was not measured in pregnancy (Table 1).

#### Sociodemographic and psychosocial variables

To investigate the social factors potentially underlying participants' perceptions of their neighbourhoods, we selected variables based on the literature and data availability; more details on measurement are included in S1 Appendix. We used the following variables reported by mothers at baseline: household education (no school leaving qualifications to postsecondary degree), marital status (married versus not), household occupational social class (manual to professional occupations), the sum total of household difficulties affording food, clothing, heating, accommodation, or items for child(ren), mothers' race/ethnicity (non-white versus not), residential mobility (mothers had moved house since becoming pregnant), depressive symptoms (Edinburgh Post-Natal Depression Scale,[48] α = .85), and social support (ALSPAC Social Network Index, α = .79).

#### Analysis plan

We used group-based trajectory modelling to estimate and compare participants' latent trajectories of objective neighbourhood deprivation exposure to their trajectories on each perceived measure of neighbourhood disadvantage using the *traj* plugin (Stata 16.0).[49,50] Group-based trajectory modelling, also known as latent class growth analysis, is a type of finite mixture modelling that aims to minimize heterogeneity within trajectory groups (random effects are not included) to approximate distinctive sections of the unknown population distribution of trajectories. This method allowed us to characterize patterns of within-participant change on both binary and scale outcomes over time (using a logit or top- and bottom-censored normal model, respectively)–it is therefore a person (as opposed to variable) centred analytic approach. Using the method's extension, dual trajectory analysis, we were able to estimate the joint distribution of the trajectory groups for objective neighbourhood deprivation and each of the perceived neighbourhood measures and the conditional probabilities of group membership (a one-step model, which accounts for misclassification bias).[49,50]

We first determined the appropriate number and shape of trajectory groups for each neighbourhood measure in univariate models based on the Bayesian Information Criterion, strength of the parameter estimates, and substantive contribution of each additional group. We considered additional model diagnostics (e.g., average posterior probabilities) according to standard best practice,[49] full details of which are in S2 Appendix. Once established, we used the parameters from the single trajectory group models to run dual trajectory models with the objective neighbourhood measure and each perceived neighbourhood measure. Parameters were estimated by maximum likelihood, which are unbiased as long as data are missing at random (i.e., the likelihood of being missing is related to the observed data but not the missing values themselves). We further excluded participants from the trajectory analyses if they did not have at least three time points on each neighbourhood variable to improve classification.[51] Models remained robust when we allowed more or less missing data, but diagnostics worsened or less common trajectory groups became more variable, respectively. We ran two additional sensitivity analyses to test the robustness of our trajectory groups: (a) using a stricter dichotomisation of neighbourhood deprivation as discussed above and (b) excluding the age 18 time point for the perceived neighbourhood measures, given the data gap preceding this (see Table 1). In all analyses we accounted for unequal time intervals by modelling the time of measurement as the age of participants’ children.

To determine which socioeconomic and psychosocial factors were correlated with the different trajectories of neighbourhood perceptions we conducted multinomial logistic regression analyses. For each perceived neighbourhood variable, trajectory group membership was regressed onto mothers' baseline covariates, controlling for baseline objective neighbourhood deprivation exposure. Using baseline data followed best practice on avoiding temporal overlap.[49] We estimated covariate associations with trajectory group membership using the modal maximum likelihood three-step method, a well-established method for accounting for potential misclassification bias which has the benefit of not subsequently altering the underlying measurement model (i.e., the trajectory groups).[52,53] We derived the matrix of classification probabilities for the trajectory groups on each perceived neighbourhood measure (a quantification of measurement error) in Stata and then exported trajectory groups to Mplus 8.3 to derive the bias-adjusted logit coefficients (currently not available in Stata).

## Results

As shown in Table 2, most mothers identified as white (98%). At baseline, 11% of mothers had moved since becoming pregnant. Most mothers (or their partners) had some secondary school qualifications (85%) and most were married (80%). Just over half of mothers’ (or their partners’) occupations were professional, managerial, or technical. Mothers' average financial difficulties score was 2.63 (SD = 3.38), with 60% experiencing any financial difficulty at baseline. Their mean depressive symptoms score was relatively low at 6.54 (SD = 4.62) (clinical cut-off is 13) and on average they had strong social networks.

**Table 2:** Sociodemographic and psychosocial characteristics of the sample overall.

	N responses	Possible range (min-max)	N (%) or M (SD)
Non-white ethnicity, N (%)	8,705	-	153 (1.76)
Recent move, N (%)	8,582	-	931 (10.85)
Household education, N (%)*	8,739	-	
No school leaving qualifications			1,270 (14.53)
Passed secondary school exams (O-level) at age 16			2,318 (26.52)
Passed secondary school exams (A-level) at age 18			3,015 (34.50)
Post-secondary degree			2,136 (24.44)
Household occupational social class, N (%)*	8,122	-	
Manual occupations			1,297 (17.11)
Skilled non-manual occupations			2,095 (27.63)
Professional, managerial, or technical occupations			4,189 (55.26)
Married, N (%)	8,757	-	7,023 (80.20)
Household financial difficulties, M (SD)	8,494	0–15	2.63 (3.38)
Depressive symptoms, M (SD)	8,157	0–30	6.54 (4.62)
Social support, M (SD)	8,531	0–30	22.54 (3.72)

Data are only presented for participants with at least three time points on all neighbourhood variables (n = 8,918).

*Highest level selected between mother and her partner.

As summarized in Table 3, the proportion of participants living in the most deprived neighbourhoods decreased over the study period, partly due to attrition and partly due to participants moving to less deprived neighbourhoods on average. Likewise, the proportion of mothers who reported a suboptimal opinion of their neighbourhood decreased over time. In contrast, mothers' experience of poor neighbourhood social cohesion improved from baseline to the age 5 assessment and worsened from the age 10 to 18 time-points. Perceived neighbourhood stress improved slightly from baseline to the age 5 time-point and then remained relatively stable.

**Table 3:** Summary of perceived and objective neighbourhood measures.

	Baseline	Age 5	Age 10	Age 18
**Objective measure**				
Exposure to more deprived neighbourhoods, N (%)	2,408 (29.72)	1,536 (24.02)	1,455 (20.94)	473 (16.38)
**Perceived measures (possible range)**				
Suboptimal opinion of neighbourhood quality, N (%)	4,807 (56.05)	3,929 (47.79)	3,075 (42.46)	1,376 (33.98)
Poor social cohesion (0–4), M (SD)	2.15 (0.57)	1.90 (0.58)	1.92 (0.55)	2.21 (0.48)
Neighbourhood stress (0–2), M (SD)	0.36 (0.32)^1^	0.30 (0.28)	0.27 (0.28)	0.29 (0.30)

Values are M (SD) or N (%) as indicated. Data are only presented for participants with at least three time points on all neighbourhood variables (n = 8,918).

^1^Measured at age 1.75 time-point.

### Trajectories of neighbourhood measures

**Fig 1:**
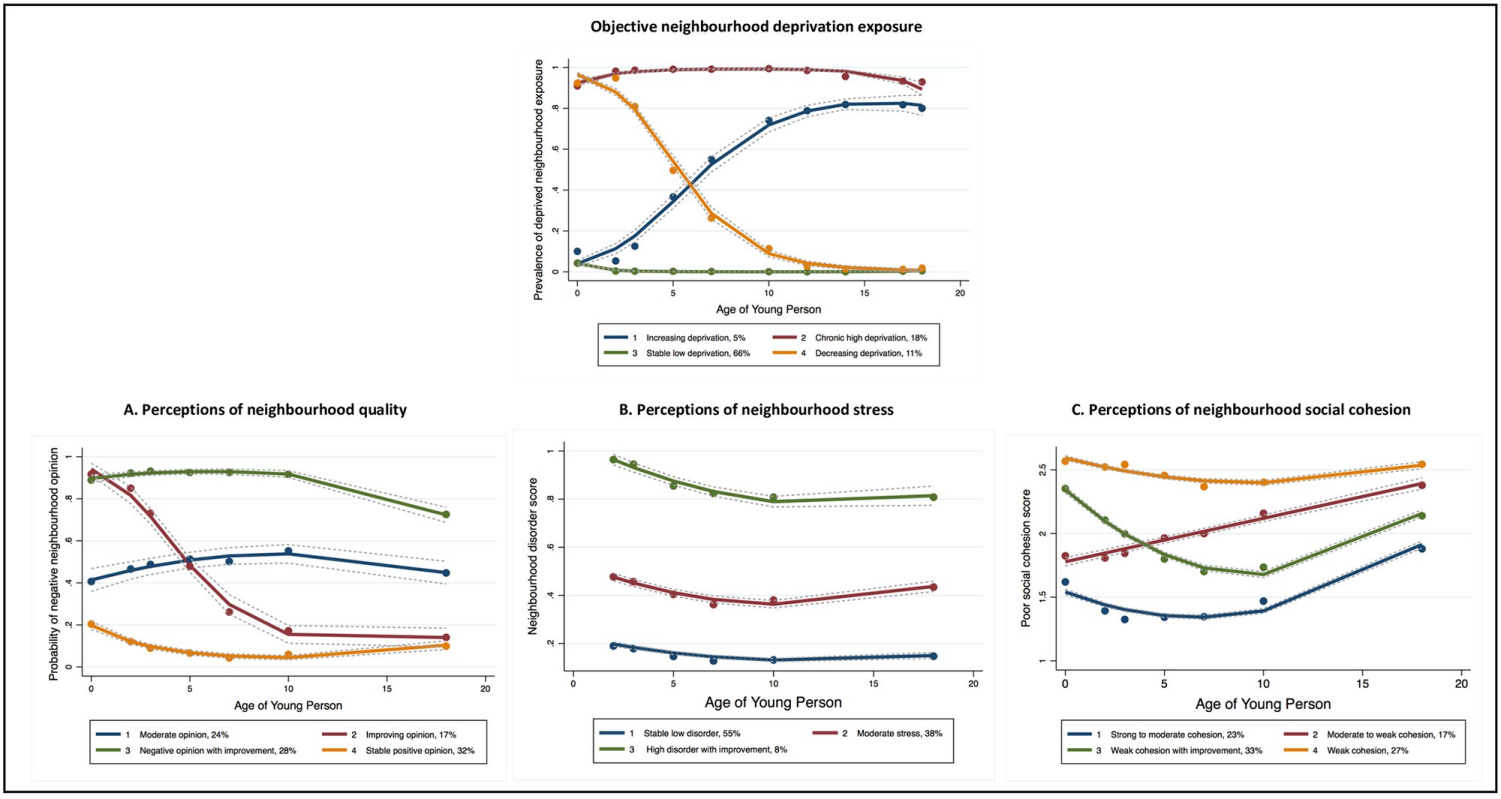
Dual trajectory models for objective neighbourhood deprivation and each perceived neighbourhood variable (N = 8,918). This figure summarizes the results from three dual trajectory analyses where the latent trajectories of objective neighbourhood deprivation exposure were estimated and compared to the latent trajectories of each of (a) perceptions of neighbourhood quality, (b) perceptions of neighbourhood stress, and (c) perceptions of neighbourhood social cohesion. The estimated trajectories for objective neighbourhood deprivation exposure are shown once for simiplicity. Solid lines are the estimated trajectories, dots are the observed group means for participating mothers at each assessment (labelled by the age of the young person participants, as per ALSPAC convention).

We first discuss the trajectories identified for each neighbourhood measure (based on the final dual trajectory analyses for consistency). This is followed by the central contribution of the dual trajectory analyses: the overlap in trajectory group membership between objective neighbourhood deprivation exposure and each perceived neighbourhood variable. Fig 1 shows the trajectory plots for each dual trajectory analysis; Table 4 provides a formal definition for each trajectory group. Parameter estimates and diagnostics for each model are included in Tables A3-A8 (S2 Appendix): all models satisfied the diagnostic criteria and entropy ranged from 0.64–0.94.

**Table 4:** Description of estimated trajectory groups by neighbourhood measure.

Trajectory group	Description
**Objective neighbourhood deprivation**	
**Stable low deprivation**	Lived consistently in more deprived neighbourhoods throughout the study period
**Increasing deprivation**	Began the study period living in less deprived neighbourhoods but then moved to more deprived neighbourhoods
**Decreasing deprivation**	Began the study period living in more deprived neighbourhoods but then moved to less deprived neighbourhoods
**Chronic high deprivation**	Lived consistently in less deprived neighbourhoods throughout the study period
**Perceived neighbourhood quality**	
**Stable positive improvement**	Held a consistently positive opinion of their neighbourhood throughout the study period
**Moderate opinion**	Held a consistently moderate opinion of their neighbourhood throughout the study period
**Improving opinion**	Began the study period with a suboptimal opinion of their neighbourhood that improved over the study period
**Negative opinion with improvement**	Held a negative opinion of their neighbourhood over most of the study period with some improvement towards the end
**Perceived neighbourhood stress**	
**Stable low stress**	Consistently perceived low neighbourhood stress throughout the study period
**Moderate stress**	Consistently perceived moderate neighbourhood stress throughout the study period
**High stress with improvement**	Perceived high neighbourhood stress over most the study period with some improvement towards the end
**Perceived social cohesion**	
**Strong to moderate cohesion**	Began the study period perceiving strong social cohesion in their neighbourhood, which weakened to perceptions of moderate social cohesion over time
**Moderate to weak cohesion**	Began the study period perceiving moderate social cohesion in their neighbourhood, which weakened to perceptions of weak social cohesion over time
**Weak cohesion with improvement**	Began the study period perceiving weak social cohesion in their neighbourhood, which improved to moderate perceptions mid-study, and then weakened again
**Weak cohesion**	Consistently perceived weak social cohesion in their neighbourhood throughout the study period

We identified four groups of participants that experienced markedly different patterns of objective neighbourhood deprivation over time (see Fig 1 and Table 4). Summarising across the dual trajectory analyses, most participants consistently lived in less deprived neighbourhoods during the study period (stable low deprivation, 66%). Nearly one-fifth of participants (18%) consistently lived in more deprived neighbourhoods (chronic high deprivation). The remaining participants experienced changing exposure over time: 11% moved to less deprived neighbourhoods over the study period (decreasing deprivation) whereas 5% moved to more deprived neighbourhoods (increasing deprivation). Our sensitivity analysis, which used a stricter dichotomisation for neighbourhood deprivation, estimated the same four trajectory groups with similar diagnostics demonstrating robustness (Tables A9-A10, S3 Appendix). The only difference was, as expected, a greater proportion of participants were assigned to the 'stable low deprivation' group under this definition.

As shown in Plot A of Fig 1, we identified four latent trajectories of mothers' opinions of their neighbourhoods. Just under one-third of participants (32%) had a stable, positive opinion of their neighbourhood over time, while 28% had a more negative opinion that improved slightly by the end of the study period. An additional 24% of participants held a moderate opinion of their neighbourhood over time. The final 17% of participants experienced a large improvement in their neighbourhood opinion during the study period.

Plot B of Fig 1 shows the three, relatively stable trajectories identified for perceived neighbourhood stress, which from most to least common were: perceptions of low stress (55%), moderate stress (38%), and high stress with improvement over the study period (8%). Plot C shows the four trajectory groups identified for poor social cohesion. The largest group (33%) experienced weak social cohesion with a period of improvement mid-study. The next most common trajectories were consistently weak social cohesion (27%) and social cohesion that progressed from strong to moderate levels (23%). The final group of participants experienced moderate to weak social cohesion over the study period (17%). All four trajectory groups experienced a near-parallel worsening in perceived social cohesion from the age 10 to age 18 time points. Our sensitivity analysis, which excluded the age 18 time point, identified the same four trajectory groups (Fig A2, S3 Appendix). This suggests that the data gap between the age 10 to 18 time points did not affect the trajectory groups estimated.

#### Perceived neighbourhood quality and objective neighbourhood deprivation

Table 5 shows the probability of trajectory group membership for each perceived neighbourhood measure conditional on the trajectory group for objective neighbourhood deprivation exposure. The green cells highlight the closest corresponding groups for each pair of measures. Higher probabilities in these green cells indicate greater convergence between the objective and perceived neighbourhood measures whereas higher probabilities in the white cells suggest greater divergence.

**Table 5:** Probability of perceived measure trajectory group conditional on objective measure trajectory group.

		Exposure to objective neighbourhood deprivation			
		Stable low deprivation	Increasing deprivation	Decreasing deprivation	Chronic high deprivation
A. Perceved neighbourhood quality	Stable positive opinion	.44	.09	.08	.05
	Moderate opinion	.24	.45	.15	.22
	Improving opinion	.15	.14	.47	.07
	Negative opinion with improvement	.15	.32	.30	.66
	Total	1.00	1.00	1.00	1.00
B. Perceived neighbourhood stress	Stable low disorder	.65	.46	.46	.23
	Moderate disorder	.32	.46	.45	.52
	High disorder with improvement	.03	.07	.10	.24
	Total	1.00	1.00	1.00	1.00
C. Perceived neighbourhood social cohesion	Strong to moderate cohesion	.28	.10	.15	.14
	Moderate to weak cohesion	.17	.20	.14	.20
	Weak cohesion with improvement	.34	.31	.34	.27
	Weak cohesion	.21	.39	.37	.39
	Total	1.00	1.00	1.00	1.00

Columns for each perceived measure total 1 as indicated. N = 8,918 participants who had at least three time points of data on all neighbourhood variables.

Overall, holding more negative opinions of neighbourhood quality was related to greater exposure to objective neighbourhood deprivation over time. As shown in Panel A of Table 5, nearly half (or more) of members of each trajectory group for objective neighbourhood deprivation tended to also be members of the closest corresponding trajectory group for neighbourhood quality. That is: 44% of participants who experienced stable low deprivation had stable positive opinions of their neighbourhoods; 45% of participant exposed to increasing neighbourhood deprivation over time reported moderate opinions of their neighbourhoods; 47% of participants exposed to decreasing deprivation reported improving opinions of their neighbourhoods; and 66% of participants exposed to chronic high deprivation held more negative opinions of their neighbourhoods. There was clear divergence between the measures as well, as evidenced by the substantial proportion of participants in each trajectory group of objective neighbourhood deprivation exposure who experienced diverging perceptions of their neighbourhood. For instance, among participants whose exposure to objective neighbourhood deprivation *increased* over time, 30% had negative opinions that, conversely, *improved* over the study period.

#### Perceived neighbourhood stress and objective neighbourhood deprivation

Similar to the results for neighbourhood quality, perceived neighbourhood stress showed a positive relationship with objective neighbourhood deprivation over time but with notable areas of divergence. Panel B in Table 5 shows that most participants exposed to stable low neighbourhood deprivation perceived stable low neighbourhood stress (65%). A large proportion of participants exposed to increasing or decreasing objective neighbourhood deprivation over the study period perceived moderate neighbourhood stress over time (47% and 45%, respectively)–most of the remaining participants in each group perceived low stress in their neighbourhoods (46% and 45%, respectively). The clear exception to this convergence was for the chronic high neighbourhood trajectory group, only 24% of whom perceived high stress in their neighbourhoods over time. Instead, most of these participants perceived moderate stress (52%), with the remaining one-quarter perceiving low stress.

#### Perceived social cohesion and objective neighbourhood deprivation

In contrast to perceived neighbourhood quality and stress, the results in Panel C of Table 5 suggest a weaker relationship between objective neighbourhood deprivation and perceived social cohesion. While there was some evidence for a negative relationship between greater neighbourhood deprivation and perceptions of weaker social cohesion, the comparatively lower probabilities of membership in the converging trajectory groups demonstrate substantial divergence between the two measures. For instance, a considerable proportion of participants who consistently lived in less deprived neighbourhoods over the study period had strong to moderate perceptions of neighbourhood social cohesion over time (28%). However, most of the participants in this low deprivation group perceived weak social cohesion with some improvement over time (34%). Moreover, the patterning of participants' perceptions of social cohesion was very similar in each of the three remaining objective deprivation groups (increasing deprivation, decreasing deprivation, and chronic high deprivation): most participants in each of these groups perceived weak social cohesion over time (ranging from 37–39%), followed by weak social cohesion with improvement (27–34%), moderate to weak social cohesion (14–20%), and strong to moderate perceptions over time (10–15%). This similarity in perceptions of social cohesion across the objective neighbourhood deprivation groups further weakens the evidence for the correlation between these two measures over time.

### Sociodemographic and psychosocial factors associated with trajectories of neighbourhood perceptions

There was a clear social patterning in the trajectories of mothers' perceptions of their neighbourhood environments, even after controlling for objective neighbourhood deprivation at baseline (Table 6). Mothers who were married or had more depressive symptoms, greater financial difficulties, or less social support at baseline tended to hold more negative opinions of their neighbourhoods over time (across all trajectory groups compared to stable positive opinions). Those who were part of households in the highest occupational social classes (professional, managerial, or technical occupations) held moderate or negative opinions of their neighbourhoods less often than positive. Participants whose households had increasingly higher educational qualifications (A-level to degree) held negative opinions of their neighbourhoods less often that positive.

**Table 6:** Covariates associated with trajectory group membership for perceived measures of neighbourhood disadvantage.

	Log odds (95% CI)	p	Log odds (95% CI)	p	Log odds (95% CI)	p
	**Prevalence of suboptimal neighbourhood opinion (referent: stable positive opinion)**					
	**Moderate**		**Improving**		**Negative opinion with improvement**	
**Depressive symptoms**	0.04 (0.01, 0.07)	0.004	0.03 (0.00, 0.06)	0.013	0.08 (0.06, 0.10)	< .001
**Recent move**	-0.05 (-0.47, 0.38)	0.828	0.12 (-0.27, 0.51)	0.535	-0.02 (-0.32, 0.28)	0.904
**Household education, referent: no school leaving qualifications**						
** Passed secondary school exams at age 16 (O-level)**	0.02 (-0.38, 0.42)	0.922	0.15 (-0.28, 0.58)	0.480	-0.14 (-0.44, 0.16)	0.356
** Passed secondary school exams at age 18 (A-level)**	-0.24 (-0.63, 0.15)	0.237	-0.11 (-0.54, 0.32)	0.607	-0.31 (-0.65, 0.04)	0.045
** Post-secondary degree**	-0.27 (-0.72, 0.18)	0.229	0.36 (-0.11, 0.83)	0.136	-0.43 (-0.69, -0.17)	0.015
**Married**	-0.46 (-0.83, -0.09)	0.015	-0.95 (-1.26, -0.64)	< .001	-0.94 (-1.77, -0.11)	< .001
**Non-white ethnicity**	0.46 (-0.50, 1.42)	0.353	0.11 (-0.81, 1.03)	0.823	0.04 (-0.24, 0.33)	0.916
**Household occupational social class, referent: manual**						
** Skilled non-manual**	-0.24 (-0.61, 0.13)	0.200	0.05 (-0.35, 0.45)	0.801	-0.21 (-0.50, 0.08)	0.147
** Professional, managerial, or technical**	-0.43 (-0.78, -0.07)	0.016	0.00 (-0.39, 0.39)	0.993	-0.32 (-0.59, -0.05)	0.020
**Social network index**	-0.05 (-0.08, -0.01)	0.007	-0.07 (-0.10, -0.04)	< .001	-0.09 (-0.12, -0.07)	< .001
**Financial difficulties**	0.12 (0.08, 0.17)	< .001	0.13 (0.08, 0.17)	< .001	0.18 (0.14, 0.21)	< .001
	**Poor social cohesion score (referent: strong to moderate cohesion)**					
	**Moderate to weak cohesion**		**Weak cohesion with improvement**		**Weak cohesion**	
**Depressive symptoms**	0.01 (-0.02, 0.04)	0.654	0.01 (-0.01, 0.03)	0.564	0.02 (0.00, 0.04)	0.117
**Recent move**	-0.25 (-0.78, 0.28)	0.352	0.19 (-0.13, 0.51)	0.244	0.19 (-0.13, 0.50)	0.253
**Household education, referent: no school leaving qualifications**						
** Passed secondary school exams at age 16 (O-level)**	-0.14 (-0.58, 0.30)	0.520	-0.05 (-0.41, 0.31)	0.780	0.00 (-0.34, 0.33)	0.984
** Passed secondary school exams at age 18 (A-level)**	-0.36 (-0.78, 0.06)	0.096	-0.42 (-0.77, -0.06)	0.022	-0.39 (-0.72, -0.06)	0.019
** Post-secondary degree**	-0.85 (-1.34, -0.36)	0.001	-0.66 (-1.04, -0.28)	0.001	-1.07 (-1.44, -0.71)	< .001
**Married**	-0.13 (-0.50, 0.24)	0.479	-0.67 (-0.94, -0.41)	< .001	-0.66 (-0.92, -0.41)	< .001
**Non-white ethnicity**	0.44 (-0.57, 1.44)	0.393	-0.14 (-1.03, 0.76)	0.767	0.43 (-0.36, 1.22)	0.289
**Household occupational social class, referent: manual**						
** Skilled non-manual**	-0.01 (-0.40, 0.38)	0.976	0.51 (0.19, 0.84)	0.002	0.43 (0.13, 0.73)	0.005
** Professional, managerial, or technical**	-0.29 (-0.65, 0.07)	0.119	0.09 (-2.97, 3.15)	0.566	-0.02 (-0.30, 0.26)	0.880
**Social network index**	-0.15 (-0.18, -0.11)	< .001	-0.12 (-0.15, -0.09)	< .001	-0.24 (-0.27, -0.21)	< .001
**Financial difficulties**	0.05 (0.01, 0.08)	0.017	-0.01 (-0.05, 0.02)	0.436	-0.01 (-0.04, 0.02)	0.536
	**Neighbourhood stress score (referent: stable low stress)**					
	**Moderate stress**		**High stress with improvement**			
**Depressive symptoms**	0.06 (0.05, 0.08)	< .001	0.12 (0.10, 0.15)	< .001		
**Recent move**	-0.17 (-0.42, 0.08)	0.200	0.07 (-0.34, 0.47)	0.739		
**Household education, referent: no school leaving qualifications**						
** Passed secondary school exams at age 16 (O-level)**	-0.07 (-0.31, 0.17)	0.564	-0.09 (-0.46, 0.28)	0.630		
** Passed secondary school exams at age 18 (A-level)**	-0.16 (-0.40, 0.08)	0.180	-0.06 (-0.45, 0.33)	0.755		
** Post-secondary degree**	0.33 (0.05, 0.59)	0.017	0.33 (-0.18, 0.84)	0.201		
**Married**	-0.22 (-0.41, -0.03)	0.021	0.60 (0.31, 0.89)	< .001		
**Non-white ethnicity**	0.35 (-0.24, 0.94)	0.240	0.99 (0.22, 1.76)	0.011		
**Household occupational social class, referent: manual**						
** Skilled non-manual**	-0.13 (-0.36, 0.10)	0.266	-0.50 (-0.84, -0.15)	0.005		
** Professional, managerial, or technical**	-0.17 (-0.39, 0.05)	0.126	-0.49 (-0.85, -0.13)	0.007		
**Social network index**	-0.04 (-0.06, -0.02)	< .001	-0.09 (-0.13, -0.06)	< .001		
**Financial difficulties**	0.12 (0.10, 0.15)	< .001	0.20 (0.17, 0.24)	< .001		

Each panel is a multinomial logistic regression where the outcome is trajectory group membership and the regressors, all measured at baseline, are the socio-demographic and psychosocial covariates (shown in the first column of the table) and exposure to objective neighbourhood-level deprivation. N = 6,247 participants who had at least three time points of data on all neighbourhood variables and baseline covariate data.

Participants who had stronger social support and were part of households with increasingly higher qualifications (A-level to degree) tended to perceive weaker social cohesion (across all trajectory groups) less often than strong to moderate social cohesion in their neighbourhoods over time. Those who were married at baseline belonged to the weakest social cohesion groups (weak cohesion and weak cohesion with improvement) less often than the stronger cohesion group. Participants whose households were in skilled non-manual compared to manual occupations more often experienced weaker rather than strong social cohesion in their neighbourhoods. Those with more financial difficulties at baseline tended to hold perceptions of social cohesion that progressed from moderate to weak over the study period rather than perceptions that progressed from strong to moderate.

Participants with more depressive symptoms and financial difficulties at baseline tended to perceive moderate or high neighbourhood stress over time as compared to low neighbourhood stress. Participants with stronger social support, who were married at baseline, and were part of increasingly higher occupational social classes (non-manual to professional occupations) tended to perceive fewer stressors in their neighbourhoods. In contrast, those who had (or whose partners had) degrees tended to perceive moderate neighbourhood stress more often than low stress. Finally, participants who were non-white tended to perceive high neighbourhood stress more often than low neighbourhood stress.

## Discussion

This study investigated the dynamic relationship between an objective measure of neighbourhood deprivation and different perceived neighbourhood disadvantages over time. While the majority of participants consistently lived in less deprived neighbourhoods (66%), the remainder experienced increasing (5%), decreasing (11%), or chronically high (18%) deprivation exposure over time–differences that would have been masked had we only considered average values of neighbourhood deprivation exposure. Likewise, we found diverse patterns of each of perceived social cohesion, neighbourhood stress, and neighbourhood quality, which diverged from participants' objective neighbourhood trajectories to varying degrees depending on the perceived measure and time point.

Our findings add to the growing literature on how exposure to and experiences of neighbourhoods vary over time and the importance of accounting for the duration and timing of exposures when determining effects on health and wellbeing.[5–7] The appropriateness of using different measures of economic or relational neighbourhood disadvantages (e.g., neighbourhood deprivation or social cohesion) as proxies for each other, or as mediators in different neighbourhood mechanisms, thus depends on the time-point (in cross-sectional studies) or period of life course (in longitudinal studies) under study and the constructs of interest. Our study further extends prior neighbourhood trajectory analyses,[54,55] which have only considered time-variation in either perceived or objective neighbourhood measures. One implication is that our results highlight the potential for interactive pathways in longitudinal neighbourhood effects: for instance, more negative trajectories across the different objective and perceived neighbourhood disadvantages studied may cumulatively predict poor health outcomes. Following a trajectory of chronic neighbourhood deprivation may similarly be offset by perceiving a cohesive neighbourhood social environment over the life course.

Overall, we found that participants’ trajectories of exposure to objective neighbourhood deprivation were negatively related to their perceptions of social cohesion; however, this relationship was the weakest of all observed. Although prior studies have found that weaker social cohesion is related to neighbourhood deprivation,[13,56] researchers have also recognized that these two constructs do not inevitably co-exist in neighburhoods.[11,57,58] Our findings add to this caution and altogether this evidence has three implications. First, it should not be assumed that people living in more deprived neighbourhoods experience low social cohesion (or that people in non-deprived neighbourhoods experience more cohesion). Neighbourhood effects on health and behaviour may require or be exacerbated by the presence of both objective deprivation and weak social cohesion. Researchers should articulate their hypothesized neighbourhood mechanisms before selecting one (or both) of these constructs as their exposure(s). Second, people’s movement across neighbourhoods can independently affect their objective exposures and their experience of those neighbourhoods (e.g., social interactions with neighbours). This is especially important to consider in the context of studies that measure neighbourhood exposures once without accounting for movement. Third, when health effects of either neighbourhood deprivation or social cohesion are observed, hypothesized mechanisms involving either construct should be explicitly tested rather than assumed. While many studies of objective neighbourhood deprivation and outcomes related to health and behaviour use social disorganization and collective efficacy theories[2,13,59]–centred on social cohesion–to explain effect mechanisms, our results demonstrate the importance of considering other potential pathways. These may include, for instance, resource access or availability, psychological stress, and social norms.[1,3,60]

Participants' perceptions of their neighbourhoods tended to be negatively correlated with individual-level socioeconomic and psychosocial markers. This likely partly underlies the differences we observed between the perceived and objective measures of neighbourhood disadvantages. Extending prior studies, we found that participants who were unmarried and had greater depressive symptoms, lower socioeconomic status, and weaker social networks at baseline tended to hold more negative perceptions of their neighbourhoods over time (weaker social cohesion, greater stress, and poorer quality)–over and above their exposure to objective neighbourhood deprivation.[20,22–25] This demonstrates the inextricability between the neighbourhood and the individual in self-reported neighbourhood measures and the need for careful analytic consideration and robustness checks (e.g., to avoid same-source bias) if associations with self-reported outcomes are of interest.[19,61]

Finally, across all of the latent trajectory groups for perceived social cohesion, there was a near-parallel decline in mothers' perceptions from when their children were 10 to 18 years old. Although inferences are limited here due to the gap in mothers’ self-reported data, this may have been due to children getting older and there resultantly being fewer opportunities for mothers to socialize within the neighbourhood (e.g., the 'empty nest' transition).[62] This suggests a unique life course component to the experience of perceived social cohesion, highlighting the especial importance of measurement timing when evaluating effects of the relational neighbourhood environment.[5–7]

### Limitations

Without direct access to participants' address data (e.g., postcodes), we were not able to investigate neighbourhood-level aggregates of the perceived meaures. Aggregated neighbourhood perceptions are less biased (although not entirely so) by individual-level factors[11,19,61]–although these factors were of interest in the current study. Having postcode data would have also allowed us to examine alternative measures of objective neighbourhood deprivation (e.g., the Townsend index) or area boundaries over time.[63] Investigating the robustness of our conclusions to these differing measures is a valuable direction for future research.

We used one iteration of the Indices of Multiple Deprivation (2010) rather than multiple iterations over time. This ensured that changes in scores reflected changes in participants' deprivation exposure (i.e., by moving neighbourhoods) and not measurement differences, but it means that we could not account for changes in neighbourhoods over time. Nonetheless, changes in relative neighbourhood deprivation in the study area were minimal over the study period–especially when considering neighbourhoods transitioning from the most deprived quintiles (4 and 5) to the least (1–3).[64] Therefore, if people's perceptions of their neighbourhoods are driven (at least partly) by relative comparisons to other neighbourhoods, this is unlikely to explain away the differences observed. In addition, a previous study of ALSPAC data which used the 2004 Indices of Multiple Deprivation identified the same four trajectory groups, demonstrating some robustness in our findings.[55]

We did not have access to participants' continuous deprivation scores (e.g., for the income or employment domains), which would have allowed us to analyze greater heterogenity and may have partly driven some of the observed divergence with the perceived measures. We favoured an analysis of neighbourhood deprivation exposure as a dichotomized rather than quintiled variable to conceptualize a threshold of more severe neighbourhood deprivation.[2] This further ensured that our trajectory groups would be parsimonious and substantively meaningful–an important quality marker for trajectory analysis.[50] Although limited in statistical power, our sensitivity analyses demonstrated that the trajectory groups identified for objective deprivation exposure were robust to a stricter dichotomisation of deprivation. We were further limited in the variability in the sample on participants’ perceptions of neighbourhood quality. Future research should test whether similar longitudinal patterns are found for perceived and objective meaures of neighbourhood disadvantage in a more diverse sample. The ALSPAC sample is predominantly white with higher socioeconomic status than the national population–generalisability to other contexts and intersectional analyses (which we were underpowered for) should be explored. Finally, results are correlational and we do not asssume a causal interpretation.

### Conclusions

Despite its limitations, this study used 18 years of robust neighbourhood and social data to interrogate the measurement of neighbourhood environments over time. We found consistent areas of convergence and divergence between trajectories of perceived and objective neighbourhood disadvantages and a distinctive patterning in neighbourhood perceptions based on individual-level social factors. Perceived measures of neighbourhood disadvantage, and especially measures of neighbourhood social processes such as social cohesion, should be considered distinct constructs from objective neighbourhood deprivation. Researchers of neighbourhood effects on health must carefully consider their hypothesized mechanism(s) when determining the most appropriate measures of neighbourhood disadvantage–and the level (neighbourhood or individual), timing, and duration for which these should be measure–to maximize relevance to theory and intervention development. Future studies that consider the outcomes of interactions between exposure pathways on different perceived and objective constructs of neighbourhood disadvantage would enrich our understanding of the potential longitudinal effects of neighbourhoods on health.

## Supporting information

S1 Appendix Measures. (PDF)

S2 Appendix Model diagnostics. (PDF)

S3 Appendix Sensitivity analyses. (PDF)
