# Supplementary material for: How do perceived and objective measures of neighbourhood disadvantage vary over time? Results from a prospective-longitudinal study in the UK with implications for longitudinal research on neighbourhood effects on health
Source: PLoS One. 2020 Apr 16;15(4):e0231779. doi: 10.1371/journal.pone.0231779 (PMC7162465; doi:10.1371/journal.pone.0231779)
Supplement: S1 Appendix — (PDF) [file pone.0231779.s001.pdf]

S1 Appendix: Measures

Table A1: 2010 English Indices of Deprivation[1]

Domain	Indicators	Weight in Index
Income deprivation	Adults and children in Income Support families;<br>Adults and children in income-based Jobseeker's Allowance families;<br>Adults and children in income-based Employment and Support Allowance families;<br>Adults and children in Pension Credit (Guarantee) families;<br>Adults and children in Child Tax Credit and Working Tax Credit families, below 60% median income not already counted;<br>Asylum seekers in England in receipt of subsistence support, accommodation support, or both.	22.5%
Employment deprivation	Claimants of Jobseeker's Allowance, aged 18-59/64;<br>Claimants of Employment and Support Allowance, aged 18-59/64;<br>Claimants of Incapacity Benefit, aged 18-59/64;<br>Claimants of Severe Disablement Allowance, aged 18-59/64;<br>Claimants of Carer's Allowance, aged 18-59/64.	22.5%
Education, skills, and training deprivation	Children and young people:<br>Key stage 2 attainment: average points score;<br>Key stage 4 attainment: average points score;<br>Secondary school absence;<br>Staying on in education post 16;<br>Entry to higher education.<br>Adults skills:<br>Adults with no or low qualifications, aged 25-59/64;<br>English language proficiency, aged 25-59/64.	13.5%
Health deprivation and disability	Years of potential life lost;<br>Comparative illness and disability ratio;<br>Acute morbidity;<br>Mood and anxiety disorders.	13.5%
Crime	Recorded crime rates for: Violence; Burglary; Theft; Criminal damage.	9.3%
Barriers to housing and services	Geographical barriers:<br>Road distance to: post office, primary school, general store/supermarket, GP surgery.<br>Wider barriers:<br>Household overcrowding;<br>Homelessness;<br>Housing affordability.	9.3%
Living environment deprivation	Indoor living environment:<br>Housing in poor condition;<br>Houses without central heating.<br>Outdoor living environment:<br>Air quality;<br>Road traffic accidents.	9.3%

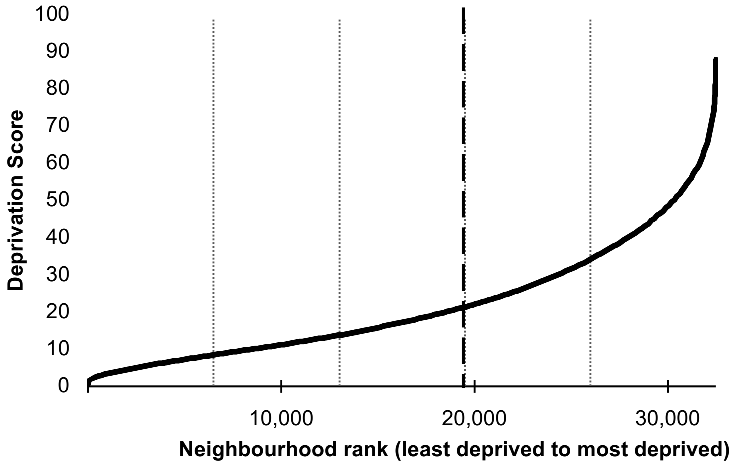

The fine dashed lines indicate quintile markers. Neighbourhoods to the right of the thick dashed line are the 40% most deprived neighbourhoods in England. As can be seen, the slope of the deprivation scores is greater in quintiles 4 and 5 versus quintiles 1-3: this represents the fact that the differences in deprivation scores between neighbourhoods in quintiles 4 and 5 are larger than the differences between neighbourhoods in quintiles 1-3. Data are from the official English Indices of Multiple Deprivation 2010 (available at: https://www.gov.uk/government/statistics/english-indices-of-deprivation-2010); further details on the measure and its construction are available in the English Indices of Multiple Deprivation 2010 technical report.[1]

Figure A1: Exponential Distribution of 2010 Indices of Multiple Deprivation Scores by Neighbourhood (Lower-Layer Super Output Area) Rank.

Table A2: Items Used in Composite Perceived Neighbourhood Variables

	**Poor social cohesion score (0-4)**
1	Neighbourhood people visiting home
2	Neighbourhood people argue with mum
3	Neighbourhood people look after mums CH
4	Neighbourhood people keep to themselves
5	Mum visits neighbours home
6	Mum argues with neighbours
7	Mum looks after neighbours CH
	**Neighbourhood stress score (0-2): until age 10**
1	Badly fitted doors and windows*
2	Poor ventilation*
3	Noise in rooms of home*
4	Noise from other homes
5	Noise from outside
6	Rubbish dumped in neighbourhood
7	Dog dirt on pavement is problem
8	Worry about vandalism
9	Worry about burglaries
10	Worry about attacks
11	Disturbance from youths
	**Neighbourhood stress score (0-2): age 18**
1	Noise from other homes
2	Noise from outside in the street
3	Rubbish or litter dumped around neighbourhood
4	Dog dirt on pavement/walkways
5	Worry about vandalism
6	Worry about burglaries
7	Worry about muggings or attacks
8	Disturbance from teenagers or youths
9	Traffic**
10	Parking**

*Item removed at age 18
**Item added at age 18

Sociodemographic and psychosocial factors

Variables were selected based on the literature and data availability in ALSPAC. We used the following sociodemographic and psychosocial variables reported by mothers at baseline. Marital status was coded as 1=married, 0=not married. Household education was coded at the highest level among the mother or her partner based on the UK education system relevant to participants at the time, where 0=no school leaving qualifications (none, Certificate of Secondary Education, or vocational), 1=O-level (secondary school exam passed at age 16), 2=A-level (secondary school exam passed at age 18), or 3=post-secondary degree. Household occupational social class was coded at the highest level among the mother or her partner based on the 1991 UK standard occupational classification, where 0=manual occupations (unskilled, partly skilled, or skilled manual), 1=skilled non-manual occupations, and 2=professional, managerial, or technical occupations. Mothers' race/ethnicity was dichotomised as 1=non-white, 0=white due to the lower proportion of non-white race/ethnicities (e.g., Asian, black) in the sample. Depressive symptoms were measured using the Edinburgh Post-Natal Depression Scale,[2] a 10-item scale which asks about the frequency of positive and negative behaviours/emotions in the last seven days (e.g., 'I have been anxious or worried for no good reason') on a 4-point scale from 0=never to 3=often. Items were all coded in the negative direction and summed so that higher scores indicate more depressive symptoms (α=.85). Residential mobility was measured based on mothers' reports of whether they had recently moved house before the baseline assessment (since becoming pregnant). Social support was measured using a Social Network Index developed for ALSPAC, where participants reported on 10 social situations (e.g., 'How many of your relatives or your partner's relatives do you see at least twice a year?'). Response categories were 0=none to 3=more than 4 and items were summed so that higher scores indicated a stronger social network (α=.79). Participants reported on their difficulty in affording each of five items – food, clothing, heating, accommodation, or items for their child(ren) – on a 4-point scale from 0=not difficult to 3=very difficult. Responses were summed to create a composite score for financial difficulties.
