# Supplementary material for: How do perceived and objective measures of neighbourhood disadvantage vary over time? Results from a prospective-longitudinal study in the UK with implications for longitudinal research on neighbourhood effects on health
Source: PLoS One. 2020 Apr 16;15(4):e0231779. doi: 10.1371/journal.pone.0231779 (PMC7162465; doi:10.1371/journal.pone.0231779)
Supplement: S2 Appendix — (PDF) [file pone.0231779.s002.pdf]

S2 Appendix: Model Diagnostics

We conducted dual trajectory analyses, an extension of longitudinal latent class analysis, to estimate and compare participants' latent trajectories of exposure to neighbourhood deprivation to their trajectories on each perceived neighbourhood measure using the Stata *traj* plugin.[3,4] We used a logit or censored normal model for binary or scale outcomes, respectively. We determined the appropriate number of latent classes for each neighbourhood measure in univariate models based on the Bayesian Information Criterion (greater values indicate better fit), parameter estimates (weak estimates indicate little additional value), and the substantive contribution of each additional group (favouring a parsimonious and theoretically meaningful model). Trajectory shapes (i.e., polynomial orders) were determined from the size and precision of the parameter estimates and model fit. At both the univariate and bivariate modelling stages, we considered additional model fit diagnostics according to standard best practice: (a) average posterior probability of group membership for individuals assigned to each group ≥ .7 (i.e., the probability of belonging to a trajectory group among its group members), (b) odds of correct classification ≥ 5 (i.e., the odds of belonging to the trajectory group based on the posterior probabilities relative to the odds of actually being a group member), (c) estimated and observed probabilities of membership for each group are similar, and (d) non-overlapping confidence intervals of estimated trajectories.[3]

Tables A3-A8 describe the parameter estimates and diagnostics for each dual trajectory analysis model included in the main manuscript. All models exceeded the diagnostic criteria. As described in the paper's method section, the sample is n=8,918 for all models.

Dual trajectory analysis: Poor perceived neighbourhood quality (logit) and deprived neighbourhood exposure (binary IMD, logit model)

Table A3: Maximum Likelihood Parameter Estimates for Trajectory Groups

Group	Parameter	Estimate (SE)	p-value
**Poor perceived neighbourhood quality (logit)**			
1 Stable positive opinion	Intercept	-1.40 (0.06)	<.001
	Linear	-0.32 (0.03)	<.001
	Quadratic	0.02 (0.00)	<.001
2 Moderate opinion	Intercept	-0.35 (0.11)	.002
	Linear	0.10 (0.03)	<.001
	Quadratic	-0.01 (0.00)	<.001
3 Improving opinion	Intercept	2.77 (0.26)	<.001
	Linear	-0.69 (0.07)	<.001
	Quadratic	0.02 (0.00)	<.001
4 Negative opinion with improvement	Intercept	2.16 (0.10)	<.001
	Linear	0.14 (0.03)	<.001
	Quadratic	-0.01 (0.00)	<.001
**More deprived neighbourhood exposure (logit)**			
1 Stable low deprivation	Intercept	-3.16 (0.07)	<.001
	Linear	-0.94 (0.07)	<.001
	Quadratic	0.05 (0.00)	<.001
2 Decreasing deprivation	Intercept	3.34 (0.15)	<.001
	Linear	-0.70 (0.05)	<.001
	Quadratic	0.01 (0.00)	<.001
3 Increasing deprivation	Intercept	-3.19 (0.20)	<.001
	Linear	0.60 (0.05)	<.001
	Quadratic	-0.02 (0.00)	<.001
4 Chronic high deprivation	Intercept	2.50 (0.10)	<.001
	Linear	0.55 (0.05)	<.001
	Quadratic	-0.03 (0.00)	<.001

Table A4: Diagnostics of Models

Group (N)	Average posterior probability of group membership	Odds of correct classification	Estimated probability of group membership (observed probability)
**Negative neighbourhood opinion (logit): entropy = 0.64**			
1 Stable positive opinion (2925)	.87	28.62	.189 (.182)
2 Moderate opinion (1905)	.72	18.33	.123 (.138)
3 Improving opinion (1505)	.70	21.75	.097 (.096)
4 Negative opinion with improvement (2583)	.85	29.12	.167 (.161)
**More deprived neighbourhood exposure (logit): entropy = 0.94**			
1 Stable low deprivation (5974)	.98	103.57	.387 (.382)
2 Decreasing deprivation (891)	.95	291.02	.058 (.063)
3 Increasing deprivation (399)	.94	549.40	.026 (.028)
4 Chronic high deprivation (1654)	.94	137.37	.107 (.104)

Dual trajectory analysis: Poor social cohesion (censored normal model) and deprived neighbourhood exposure (binary IMD, logit model)

Table A5: Maximum Likelihood Parameter Estimates for Trajectory Groups

Group	Parameter	Estimate (SE)	p-value
**Poor social cohesion score (censored normal)**			
1 Strong to moderate cohesion	Intercept	1.54 (0.01)	<.001
	Linear	-0.06 (0.00)	<.001
	Quadratic	0.00 (0.00)	<.001
2 Moderate to weak cohesion	Intercept	1.78 (0.02)	<.001
	Linear	0.03 (0.00)	<.001
3 Weak cohesion with improvement	Intercept	2.34 (0.01)	<.001
	Linear	-0.14 (0.00)	<.001
	Quadratic	0.01 (0.00)	<.001
4 Weak cohesion	Intercept	2.59 (0.01)	<.001
	Linear	-0.04 (0.00)	<.001
	Quadratic	0.00 (0.00)	<.001
**More deprived neighbourhood exposure (logit)**			
1 Stable low deprivation	Intercept	-3.16 (0.07)	<.001
	Linear	-0.93 (0.07)	<.001
	Quadratic	0.05 (0.00)	<.001
2 Decreasing deprivation	Intercept	3.30 (0.14)	<.001
	Linear	-0.70 (0.04)	<.001
	Quadratic	0.01 (0.00)	<.001
3 Increasing deprivation	Intercept	-3.19 (0.20)	<.001
	Linear	0.60 (0.05)	<.001
	Quadratic	-0.02 (0.00)	<.001
4 Chronic high deprivation	Intercept	2.50 (0.11)	<.001
	Linear	0.58 (0.05)	<.001
	Quadratic	-0.03 (0.00)	<.001

Table A6: Diagnostics of Models

Group (N)	Average posterior probability of group membership	Odds of correct classification	Estimated probability of group membership (observed probability)
**Poor social cohesion score (censored normal): entropy = 0.68**			
1 Strong to moderate cohesion (2107)	.87	43.15	.136 (.134)
2 Moderate to weak cohesion (1362)	.72	26.50	.089 (.100)
3 Weak cohesion with improvement (3027)	.77	13.96	.196 (.188)
4 Weak cohesion (2422)	.89	43.40	.157 (.156)
**More deprived neighbourhood exposure (logit): entropy = 0.94**			
1 Stable low deprivation (5978)	.98	102.85	.387 (.382)
2 Decreasing deprivation (876)	.96	393.08	.057 (.064)
3 Increasing deprivation (402)	.93	494.54	.026 (.028)
4 Chronic high deprivation (1662)	.94	121.43	.108 (.103)

Dual trajectory analysis: Neighbourhood disorder (censored normal model) and deprived neighbourhood exposure (binary IMD, logit model)

Table A7: Maximum Likelihood Parameter Estimates for Trajectory Groups

Group	Parameter	Estimate (SE)	p-value
**Neighbourhood disorder (censored normal)**			
1 Stable low disorder	Intercept	0.21 (0.01)	<.001
	Linear	-0.02 (0.00)	<.001
	Quadratic	0.00 (0.00)	<.001
2 Moderate disorder	Intercept	0.53 (0.01)	<.001
	Linear	-0.03 (0.00)	<.001
	Quadratic	0.00 (0.00)	<.001
3 High disorder with improvement	Intercept	1.08 (0.02)	<.001
	Linear	-0.04 (0.00)	<.001
	Quadratic	0.00 (0.00)	<.001
**More deprived neighbourhood exposure (logit)**			
1 Stable low deprivation	Intercept	-3.15 (0.07)	<.001
	Linear	-0.92 (0.07)	<.001
	Quadratic	0.05 (0.00)	<.001
2 Decreasing deprivation	Intercept	3.36 (0.14)	<.001
	Linear	-0.71 (0.05)	<.001
	Quadratic	0.01 (0.00)	<.001
3 Increasing deprivation	Intercept	-3.25 (0.20)	<.001
	Linear	0.62 (0.05)	<.001
	Quadratic	-0.02 (0.00)	<.001
4 Chronic high deprivation	Intercept	2.47 (0.10)	<.001
	Linear	0.56 (0.05)	<.001
	Quadratic	-0.03 (0.00)	<.001

Table A8: Diagnostics of Models

Group (N)	Average posterior probability of group membership	Odds of correct classification	Estimated probability of group membership (observed probability)
**Neighbourhood disorder (censored normal): entropy = 0.79**			
1 Stable low disorder (4977)	.91	21.86	.322 (.317)
2 Moderate disorder (3291)	.87	25.45	.213 (.218)
3 High disorder with improvement (650)	.93	281.57	.042 (.043)
**More deprived neighbourhood exposure (logit): entropy = 0.94**			
1 Stable low deprivation (5979)	.98	100.92	.387 (.382)
2 Decreasing deprivation (869)	.96	384.36	.056 (.064)
3 Increasing deprivation (397)	.93	518.67	.026 (.028)
4 Chronic high deprivation (1673)	.94	122.10	.108 (.104)
