# Supplementary material for: How do perceived and objective measures of neighbourhood disadvantage vary over time? Results from a prospective-longitudinal study in the UK with implications for longitudinal research on neighbourhood effects on health
Source: PLoS One. 2020 Apr 16;15(4):e0231779. doi: 10.1371/journal.pone.0231779 (PMC7162465; doi:10.1371/journal.pone.0231779)
Supplement: S3 Appendix — (PDF) [file pone.0231779.s003.pdf]

S3 Appendix: Sensitivity Analyses

Table A9 summarizes the sensitivity analysis for objective neighbourhood deprivation exposure, where more deprived neighbourhoods were instead described as the top 20% of the most deprived neighbourhoods in the UK. As shown, the same longitudinal latent classes were derived in this sensitivity analysis as in the main analysis (only proportions differed, as expected). Table A10 shows the diagnostic criteria for this model which, as with the main analysis, were excellent.

Sensitivity analysis: Deprived neighbourhood exposure using a stricter dichotomization (1=top 20% most deprived neighbourhoods in the UK, 0=all others)

Table A9: Maximum Likelihood Parameter Estimates for Trajectory Groups (Univariate Binary Logit Model)

Group	Parameter	Estimate (SE)	p-value
1 Stable low deprivation	Intercept	-3.87 (0.09)	<.001
	Linear	-0.93 (0.09)	<.001
	Quadratic	0.05 (0.01)	<.001
2 Decreasing deprivation	Intercept	2.49 (0.19)	<.001
	Linear	0.43 (0.07)	<.001
	Quadratic	-0.01 (0.01)	.079
3 Increasing deprivation	Intercept	-3.35 (0.30)	<.001
	Linear	0.71 (0.07)	<.001
	Quadratic	-0.03 (0.00)	<.001
4 Chronic high deprivation	Intercept	1.80 (0.14)	<.001
	Linear	0.60 (0.07)	<.001
	Quadratic	-0.03 (0.00)	<.001

Table A10: Diagnostics of Model (Univariate Binary Logit Model)

Group (N)	Average posterior probability of group membership	Odds of correct classification	Estimated probability of group membership (observed probability)
1 Stable low deprivation (7706)	.99	134.47	.499 (.496)
2 Decreasing deprivation (414)	.95	747.18	.027 (.031)
3 Increasing deprivation (217)	.89	572.24	.014 (.015)
4 Chronic high deprivation (581)	.90	234.84	.038 (.035)

Sensitivity analysis: Removing the age 18 time point from the trajectory analyses of the perceived neighbourhood measures

Figure A2: Trajectory Models for Each Perceived Neighbourhood Variable Excluding the Age 18 Time Point (N=8,918)

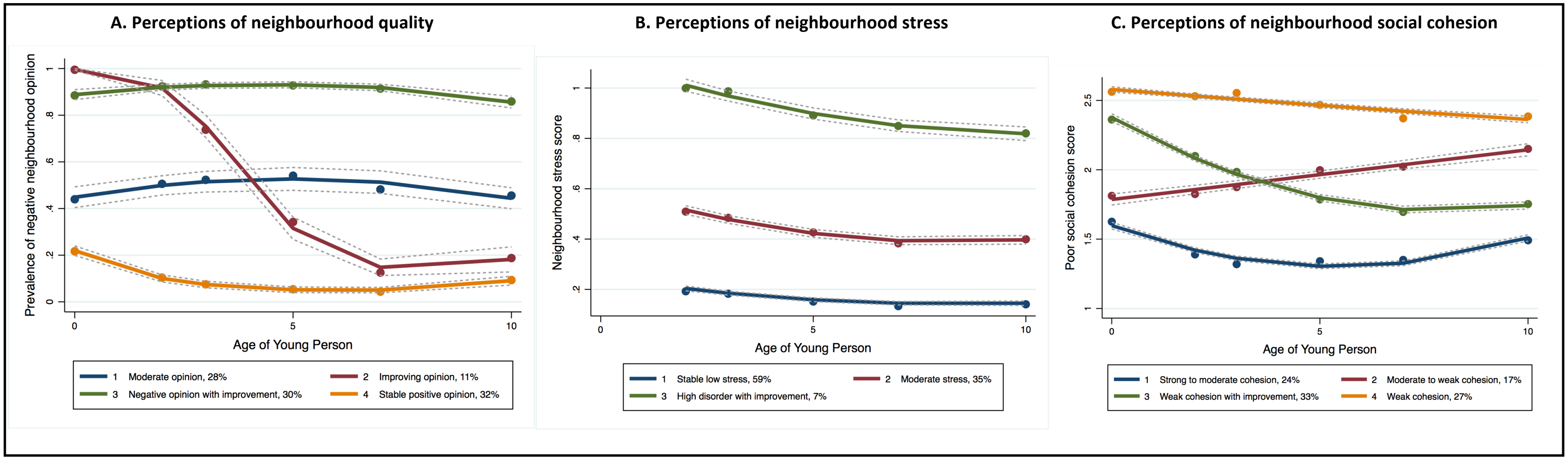

References

1 Lad M. The English Indices of Multiple Deprivation 2010. London: Department for Communities and Local Government; 2011.

2 Cox JL, Holden JM, Sagovsky R. Detection of postnatal depression: Development of the 10-item Edinburgh Postnatal Depression Scale. British Journal of Psychiatry. 1987;150:782-6.

3 Nagin DS. Group-Based Modeling of Development. London: Harvard University Press; 2005.

4 Nagin DS, Odgers CL. Group-based trajectory modeling in clinical research. Annual review of clinical psychology. 2010;6:109-38.
